# Sex and age distributions of persons in foodborne disease outbreaks and associations with food categories

**DOI:** 10.1017/S0950268818003126

**Published:** 2019-05-29

**Authors:** P. D. Strassle, W. Gu, B. B. Bruce, L. H. Gould

**Affiliations:** 1 Department of Epidemiology, Emory University, Atlanta, Georgia, USA; 2 Department of Epidemiology, University of North Carolina at Chapel Hill, Chapel Hill, North Carolina, USA; 3 Division of Foodborne, Waterborne, and Environmental Diseases, Centers for Disease Control and Prevention, Atlanta, Georgia, USA

**Keywords:** Foodborne outbreaks, epidemiology, sex differences, age differences

## Abstract

Sex and age differences in food preferences may be reflected in the demographics of outbreaks. Outbreaks from 1998–2015 with a single confirmed implicated food source in the Centers for Disease Control and Prevention Foodborne Disease Outbreak Surveillance System were analysed using logistic regression to assess associations between a food category, sex and age. Males were more likely to be involved in outbreaks attributed to beef, pork, game, dairy and shellfish; females were more likely to be involved in grains-beans, nuts-seeds, fruits, sprouts and vegetable row crops outbreaks. Children <5-years-old were more likely than other age groups to be involved in dairy outbreaks, children 5–19-years-old were most likely to be involved in beef and game outbreaks, adults 20–49-years-old were most likely to be involved in fish, shellfish and sprout outbreaks and adults ⩾50-years-old were most likely to be involved in turkey outbreaks. Age and sex are associated with specific food categories in outbreaks. This information may be useful in helping to identify sources of foodborne disease outbreaks.

## Introduction

There are an estimated 9.4 million foodborne illnesses caused by known pathogens in the USA every year [1]. Although most are sporadic, outbreak-associated illnesses are an important source of information about the foods and pathogens causing illness [2, 3]. Identifying the source of an outbreak is a critical part of containment and removal of the contaminated source from the food supply.

Several factors can affect the quantity and variety of food an individual or family consumes, including income, sex, age, race and ethnicity and health status [4–12]. For example, men are more likely to consume ‘high risk’ foods, including unpasteurised milk, raw shellfish, runny eggs and pink hamburgers than women [4–6]. This difference was also observed for school-aged boys and girls [6–8]. Women are more likely to consume fruits and vegetables, including alfalfa sprouts, a high-risk food [6]. Differences in consumption patterns between school-aged children and adults have also been shown in national surveys; children are more likely to consume raw fruits and tomatoes, and adults more likely to eat dark green vegetables, cantaloupe and strawberries [10].

Given the sex and age differences in food preferences, it remains unknown whether these differences are reflected in the demographics of outbreak-associated foodborne illness. Using data from foodborne disease outbreaks in the USA, we analysed the associations among contaminated food sources and demographics (age and sex) of outbreak-associated illnesses.

## Methods

Since 1973, the Centers for Disease Control and Prevention (CDC) has collected data on foodborne disease outbreaks (defined as two or more persons with a similar illness and exposure to a common food) through the Foodborne Disease Outbreak Surveillance System (FDOSS). FDOSS is a passive national surveillance system with state, local, territorial and federal public health agencies reporting outbreaks caused by bacterial, viral, parasitic and chemical agents [2]. Data collected for each outbreak include the implicated food, number of illnesses, reporting state, location of food preparation (e.g. restaurant), etiologic agent, the number and/or proportion of men and women, number and/or proportion of cases aged <5-years-old, 5–19-years-old, 20–49-years-old and ⩾50-years-old. Only confirmed aetiologic agents were included in analyses. Data are reported in aggregate for each outbreak and information on individual cases are not captured in the FDOSS system.

Outbreaks with first illness onset from 1 January 1998 to 31 December 2015, were reported to FDOSS by 20 December 2016, occurred outside of a correctional institute and had a single confirmed implicated food source category (as defined by the Interagency Food Safety Analytics Collaboration) were eligible for inclusion [13]. The 19 categories included beef, pork, chicken, turkey, game, dairy, eggs, fish, shellfish, oils-sugars, grains-beans, nuts-seeds, fruits, fungi, herbs, root-underground vegetables (e.g. carrots, potatoes), seeded vegetables (e.g. legumes, tomatoes), sprouts and vegetable row crops (e.g. asparagus, lettuce) [13]. Outbreaks with ‘other’ (e.g. other poultry) and nonspecific (e.g. land animals) implicated food sources were excluded. During those years, there were 18 938 outbreaks, which caused 355 239 illnesses (median 8, range 2–1939) and of those, 4008 outbreaks causing 75 845 illnesses met the inclusion criteria and were included in the analysis. The majority of excluded outbreaks (*n* = 10 345, 72%) were excluded due to not having a confirmed implicated food source. Counts of males, females and persons in each age category were calculated using reported proportions when counts were not directly reported by the health department. Overall, outbreak records were missing data on sex for 17 357 (23%) persons and were missing data on age for 29 985 (40%) persons. This missing data were assumed to be missing completely at random, and thus assumed not to bias the estimation of the relationship of sex and age on food category [14].

We calculated odds ratios using logistic regression on sex and age group (categorised as <5, 5–19, 20–49 (reference) and ⩾50-years-old) for each food category. We used contrast statements to compare individual categories of age to all other ages combined. For multiple comparison testing, we adopted a Bonferroni correction and a *P*-value < 0.003 was considered statistically significant (at the nominal significance of 0.05). All analyses were performed using SAS 9.4 (SAS Institute., Cary, NC, USA). Institutional Review Board (IRB) exemption was received from Emory University.

## Results

Among the 4008 included outbreaks, fish (21%), beef (16%), chicken (11%), shellfish (9%) and pork (7%) were the five most common implicated food categories (Table 1). Outbreak-associated illnesses were slightly more likely to be in females (54%) than males (46%). By age group, 6% of all cases were <5-years-old, 19% were between 5 and 19-years-old, 47% were between 20 and 49-years-old and 28% were ⩾50-years-old.

**Table 1.** Frequency of food categories in outbreaks with a single implicated food source and reported to FDOSS between 1998 and 2015

	Outbreaks *N* (%)	Illnesses *N* (%)
All outbreaks	4008 (100)	75 845 (100)
Fish	827 (20.6)	4696 (6.2)
Beef	641 (16.0)	10 426 (13.7)
Chicken	446 (11.1)	7945 (10.5)
Shellfish	375 (9.4)	4046 (5.3)
Pork	288 (7.2)	6392 (8.4)
Dairy	282 (7.0)	4536 (6.0)
Vegetable row crops	194 (4.8)	6131 (8.1)
Turkey	177 (4.4)	5147 (6.8)
Eggs	170 (4.2)	5597 (7.4)
Grains-beans	143 (3.6)	1358 (1.8)
Fruits	128 (3.2)	5194 (6.8)
Seeded vegetables	93 (2.3)	7115 (9.4)
Root-underground	69 (1.7)	1027 (1.4)
Sprouts	48 (1.2)	1612 (2.1)
Fungi	35 (0.9)	164 (0.2)
Herbs	31 (0.8)	2208 (2.9)
Game	28 (0.7)	196 (0.3)
Nuts-seeds	22 (0.6)	1876 (2.5)
Oils-sugars	11 (0.3)	179 (0.2)

Outbreak aetiology was identified in 59% (*n* = 2345) of outbreaks, which accounted for 76% of cases (*n* = 57 992). The most common outbreak categories with viral aetiologies (219 outbreaks) were row crops (25%), shellfish (21%) and beef (12%). Among bacterial outbreaks (1473 outbreaks), over half of all outbreaks were caused by beef (18%), chicken (13%), dairy (14%) and pork (10%) and over half of parasitic outbreaks (*n* = 43) were caused by game (*n* = 15, 35%) and fruit (*n* = 10, 23%). Almost all outbreaks caused by fish toxins (537 outbreaks) were in fish (97%), compared with shellfish (3%). Fish also made up over half (*n* = 42, 58%) of all outbreaks caused by ‘other’ aetiologies (73 outbreaks).

Males were more likely to be involved in outbreaks attributed to beef (OR 1.17, 95% CI 1.12–1.23), shellfish (OR 1.62, 95% CI 1.50–1.75), pork (1.32, 95% CI 1.25–1.39), dairy (OR 1.24, 95% CI 1.16–1.32) and game (OR 2.55, 95% CI 1.86–3.50), (Table 2). On the other hand, females were more likely to be involved in outbreaks attributed to vegetable row crops (OR 1.41, 95% CI 1.32–1.50), grains-beans (OR 1.27, 95% CI 1.12–1.44), fruits (OR 1.45, 95% CI 1.36–1.54), seeded vegetables (OR 1.10, 95% CI 1.04–1.16), sprouts (OR 1.24, 95% CI 1.10–1.40) and nuts-seeds (OR 1.26, 95% CI 1.14–1.38).

**Table 2.** Odds of being involved in an outbreak for males, compared with females, given the category in outbreaks reported to FDOSS between 1998 and 2015

	Males *N* (%)	Female *N* (%)	OR (95% CI)
All outbreaks	27 109 (46.4)	31 379 (53.7)	–
Fish	1733 (45.9)	2047 (54.2)	0.98 (0.92–1.05)
Beef	4017 (50.2)	3979 (49.8)	**1.17 (1.12–1.23)**
Chicken	2908 (46.6)	3333 (53.4)	1.01 (0.96–1.07)
Shellfish	1557 (57.8)	1137 (42.2)	**1.62 (1.50–1.75)**
Pork	2872 (52.6)	2588 (47.4)	**1.32 (1.25–1.39)**
Dairy	1981 (51.4)	1875 (48.6)	**1.24 (1.16–1.32)**
Vegetable row crops	1640 (38.6)	2612 (61.4)	**0.71 (0.67–0.76)**
Turkey	2065 (44.9)	2532 (55.1)	0.94 (0.88–1.00)
Eggs	1246 (45.1)	1520 (55.0)	0.95 (0.88–1.02)
Grains-beans	431 (40.6)	631 (59.4)	**0.79 (0.70–0.89)**
Fruits	1681 (38.0)	2741 (62.0)	**0.69 (0.65–0.74)**
Seeded vegetables	2472 (44.2)	3118 (55.8)	**0.91 (0.86–0.96)**
Root-underground	368 (44.0)	469 (56.0)	0.91 (0.79–1.04)
Sprouts	483 (41.4)	691 (58.9)	**0.81 (0.72–0.91)**
Fungi	66 (52.4)	60 (47.6)	1.27 (0.90–1.81)
Herbs	705 (47.0)	796 (53.0)	1.03 (0.93–1.14)
Game	123 (68.7)	56 (31.3)	**2.55 (1.86–3.50)**
Nuts-seeds	733 (40.9)	1059 (59.1)	**0.80 (0.72–0.88)**
Oils-sugars	66 (40.5)	97 (59.5)	0.79 (0.58–1.08)

OR, odds ratio; CI, confidence interval.

Bold indicates the significance of *P* < 0.003 (Bonferroni corrected).

As age increased, the odds of being involved in a dairy outbreak significantly decreased, with children <5-years-old about 3 times as likely (OR 3.18, 95% CI 2.85–3.54), 5–19-yearsold about twice as likely (OR 2.25, 95% CI 2.07–2.45) and adults ⩾50-years-old significantly less likely to be involved (OR 0.71, 95% CI 0.64–0.79) compared with 20–49-years-old (Table 3). To determine whether certain age categories were at the highest or lowest risk compared with those of other ages, we also tested contrasts comparing each age group to all other age groups combined. In these analyses, children <5-years-old were most likely to be involved in dairy (OR 3.18, 95% CI 2.85–3.54), fruit (OR 2.55, 95% CI 2.25–2.89) and seeded vegetable (OR 2.13, 95% CI 1.89–2.40) outbreaks and least likely to be involved in pork (OR 0.65, 95% CI 0.56–0.76), vegetable row crop (OR 0.54, 95% CI 0.45–0.66), or grain-bean (OR 0.57, 95% CI 0.40–0.81) outbreaks. Children 5–19-years-old were most likely to be involved in beef (OR 1.79, 95% CI 1.67–1.92) and game (OR 3.07, 95% CI 1.97–4.78) outbreaks. Adults 20–49-years-old were most likely to be involved in fish (OR 2.72, 95% CI 2.45–3.03), shellfish (OR 4.00, 95% CI 3.29–4.86), root-underground (OR 1.88, 95% CI 1.52–2.34) and sprout (OR 2.12, 95% CI 1.79–2.51) outbreaks and least likely to be involved in nut-seed (OR 0.42, 95% CI 0.41–0.49) outbreaks. Finally, adults ⩾50-years-old were most likely to be involved in turkey (OR 1.94, 95% CI 1.76–2.14) and herb (OR 1.51, 95% CI 1.26–1.82) outbreaks and least likely to be involved in chicken (OR 0.64, 95% CI 0.59–0.69) outbreaks. Egg, fungi and oil-sugar outbreaks had no single age group that was significantly at higher or lower odds than all other age groups combined.

**Table 3.** Odds of being involved in an outbreak among individuals <5-years-old, 5–19-years-old and ⩾50-years-old, compared with 20–49-years-old, stratified by food category in outbreaks reported to FDOSS between 1998 and 2015

	<5 years		5–19 years		20–49 years		⩾50 years	
	*N* (%)	OR (95% CI)	*N* (%)	OR (95% CI)	*N* (%)	OR (95% CI)	*N* (%)	OR (95% CI)
All outbreaks	2546 (5.6)	–	8780 (19.2)	–	21 637 (47.2)	–	12 897 (28.1)	–
Fish	63 (2.0)	**0.25** (**0.20–0.34)**	250 (8.1)	**0.29** (**0.25–0.33)**	1977 (63.6)	Ref	817 (26.3)	**0.67** (**0.62–0.73)**
Beef	289 (4.0)	**0.75 (0.66–0.85)**	1902 (26.4)	**1.61 (1.51–1.71)**	3173 (44.0)	Ref	1845 (25.6)	0.97 (0.91–1.03)
Chicken	326 (6.4)	1.07 (0.95–1.22)	1078 (21.3)	1.08 (0.95–1.11)	2591 (51.2)	Ref	1070 (21.1)	**0.67 (0.62–0.72)**
Shellfish	14 (0.6)	**0.08 (0.05–0.14)**	122 (5.5)	**0.21 (0.17–0.25)**	1356 (61.1)	Ref	729 (32.8)	0.90 (0.82–0.98)
Pork	183 (3.8)	**0.63 (0.54–0.73)**	890 (18.4)	0.91 (0.84–0.99)	2377 (49.1)	Ref	1395 (28.8)	0.98 (0.92–1.05)
Dairy	483 (14.2)	**3.71 (3.31–4.16)**	1092 (32.0)	**2.25 (2.07–2.45)**	1283 (37.6)	Ref	552 (16.2)	**0.71 (0.64–0.79)**
Vegetable row crops	113 (3.2)	**0.54 (0.45–0.66)**	604 (16.9)	**0.86 (0.78–0.95)**	1704 (47.5)	Ref	1163 (32.5)	1.16 (1.07–1.25)
Turkey	87 (2.3)	**0.38 (0.31–0.48)**	547 (14.3)	**0.72 (0.65–0.79)**	1837 (48.1)	Ref	1349 (35.3)	**1.26 (1.17–1.36)**
Eggs	135 (5.7)	1.09 (0.91–1.31)	417 (17.5)	0.97 (0.86–1.09)	1059 (44.4)	Ref	773 (32.4)	**1.24 (1.13–1.36)**
Grains-beans	33 (3.3)	0.59 (0.41–0.84)	203 (20.4)	1.07 (0.90–1.26)	470 (47.3)	Ref	287 (28.9)	1.03 (0.88–1.19)
Fruits	323 (12.4)	**3.71 (3.24–4.25)**	459 (17.6)	**1.41 (1.25–1.58)**	815 (31.2)	Ref	1014 (38.8)	**2.18 (1.98–2.40)**
Seeded vegetables	352 (10.8)	**2.61 (2.30–2.97)**	670 (20.5)	**1.35 (1.22–1.48)**	1251 (38.2)	Ref	1001 (30.6)	**1.37 (1.26–1.50)**
Root-underground	13 (1.6)	**0.25 (0.14–0.43)**	122 (14.9)	**0.67 (0.55–0.82)**	444 (54.2)	Ref	241 (29.4)	0.91 (0.78–1.06)
Sprouts	27 (3.1)	**0.40 (0.27–0.59)**	126 (14.4)	**0.55 (0.45–0.66)**	561 (64.0)	Ref	162 (18.5)	**0.48 (0.40–0.57)**
Fungi	1 (0.8)	0.13 (0.02–0.96)	14 (11.3)	0.54 (0.30–0.96)	64 (51.6)	Ref	45 (36.3)	1.18 (0.81–1.73)
Herbs	37 (4.8)	0.76 (0.54–1.06)	70 (9.0)	**0.41 (0.32–0.53)**	414 (53.5)	Ref	253 (32.7)	1.03 (0.88–1.20)
Game	4 (2.5)	0.45 (0.17–1.24)	58 (36.0)	**1.91 (1.36–2.70)**	75 (46.6)	Ref	24 (14.9)	0.54 (0.34–0.85)
Nuts-seeds	54 (12.1)	**3.43 (2.49–4.71)**	114 (25.6)	**2.08 (1.62–2.67)**	136 (30.5)	Ref	142 (31.8)	**1.76 (1.39–2.23)**
Oils-sugars	9 (6.6)	1.53 (0.75–3.12)	42 (30.9)	**2.08 (1.38–3.13)**	50 (36.8)	Ref	35 (25.7)	1.17 (0.76–1.81)

OR, odds ratio; CI, confidence interval.

Bold indicates the significance of *P* < 0.003 (Bonferroni corrected).

## Discussion

Based on an analysis of 18 years of FDOSS data, our results suggest that specific sex and age groups are associated with a higher odds of being in outbreaks from certain foods. These associations might assist in the investigation of the cause of foodborne disease outbreaks. Outbreaks associated with red meats, dairy and shellfish had more males and outbreaks associated with vegetable row crops, grains-beans, fruits, seeded vegetables, sprouts and nuts-seeds were more likely to involve females. Age distributions in outbreaks also varied by food category. Young children (<5-years-old) were most likely to be involved in outbreaks attributed to dairy, fruits and seeded vegetables and children 5–19-years-old were most likely to be involved in outbreaks attributed to beef and game. Additionally, adults 20–49 were most likely to be associated with outbreaks involving fish, shellfish and sprouts and older adults (⩾50-years-old) were most likely to be involved in outbreaks attributed to turkey. This study demonstrates that the demographic distribution of outbreak illnesses might result from the demographically related food preferences. These results are consistent with previous studies of food consumption patterns across age and sex in the USA [4–12]. It is also possible that these results reflect pathogen patterns, as foods can be associated with specific pathogens and age has been shown to be a risk factor for certain types of infections [15–17].

While this study focused on aggregated outbreak data, age and sex differences in individual outbreaks can be quite striking, making these associations potentially even more useful. For example, in a 2009 multistate outbreak (228 cases, 13 states) involving alfalfa sprouts, 69% of patients were female and the median age was 29 [18]. And in a 2014 outbreak involving raw milk in Utah (99 cases), 44% of cases were in children <18 years of age, despite this age group only making up roughly 1/4 of all outbreak cases [19]. Moreover, information on food consumption patterns has already been used to further confirm the identification of contamination sources in foodborne disease outbreaks. In a 2004 outbreak of *Salmonella* Enteritidis infections in Oregon, hypothesis-generating interview results were compared with the food consumption patterns of Oregon residents and helped identify raw almonds as the source of the outbreak [20]. In 2008, the source of an outbreak of *Salmonella* Agona infections was further confirmed by comparing the percentage of cases reported eating puffed rice cereal with the total ready-to-eat cereal market share in the USA [21]. Recently, a similar prediction tool by White *et al*. was created to assist in food category identification, specifically for Shiga toxin-producing *Escherichia coli* outbreaks [22]. They found that sex distributions predicted the implicated food- with women being more likely to be involved in vegetable outbreaks-, although their analyses were limited to outbreaks caused by beef, vegetables and dairy products. Our analyses also suggest that sex, as well as age, may help in the identification of implicated foods during outbreak investigations.

A limitation of this study is that sex and age are reported in aggregate and therefore interactions between the two variables could not be meaningfully assessed. Future studies could refine these estimates by using datasets where both the age and sex of each case is known. Additional research into more specific food groups may also be required. For example, while young children were most likely to be involved with a nut-seed outbreak, this category included outbreaks caused by whole nuts, nut butter (including peanut butter), crushed/ground seeds (e.g. chia seed powder) and seed paste (e.g. tahini). In this analysis, almost 80% of nut-seed outbreak cases were involved in a peanut butter outbreak and it is very possible that young children may not be more likely to be in outbreaks caused by all of these different nut-seed foods. There was also a high proportion of missing data which we assumed to be missing completely at random and not to be a source of bias in our results, which may not be true. Additionally, FDOSS is a dynamic surveillance system and the results of this study may differ from prior or future reports. Finally, only a small proportion of foodborne illnesses are identified as being associated with outbreaks and many outbreaks never have a food source identified and/or have unknown etiologies. In FDOSS, roughly half of all foodborne outbreaks have no implicated food source and were excluded from this analysis.

Knowledge about associations among outbreak demographic characteristics and contaminated food sources is still limited. As novel contamination routes and outbreak sources continue to become more prevalent, new methods for outbreak source identification will need to be developed. This study suggests that outbreak demographic information may be useful to assist investigators in narrowing down potential contaminated food sources in outbreaks. Additional research is needed to improve food profiling in outbreak investigations.
